# Role of *WNT10A* in failure of tooth development in humans and zebrafish

**DOI:** 10.1002/mgg3.332

**Published:** 2017-09-14

**Authors:** Qiuping Yuan, Min Zhao, Bhavna Tandon, Lorena Maili, Xiaoming Liu, Anqi Zhang, Evan H. Baugh, Tam Tran, Renato M. Silva, Jacqueline T. Hecht, Eric C. Swindell, Daniel S. Wagner, Ariadne Letra

**Affiliations:** ^1^ Department of Pediatrics University of Texas Health Science Center at Houston Medical School Houston Texas; ^2^ Pediatric Research Center University of Texas Health Science Center at Houston Medical School Houston Texas; ^3^ Center for Craniofacial Research University of Texas Health Science Center at Houston School of Dentistry Houston Texas; ^4^ Department of Biosciences Rice University Houston Texas; ^5^ Department of Human Genetics University of Texas Health Science Center at Houston School of Public Health Houston Texas; ^6^ Department of Biology New York University New York New York; ^7^ Department of Endodontics University of Texas Health Science Center at Houston School of Dentistry Houston Texas; ^8^ University of Texas Graduate School of Biomedical Sciences at Houston Houston Texas 77030; ^9^ Department of Diagnostic and Biomedical Sciences University of Texas Health Science Center at Houston School of Dentistry Houston Texas

**Keywords:** Animal model, gene, knockdown, oligodontia, whole‐exome sequencing, *WNT*

## Abstract

**Background:**

Oligodontia is a severe form of tooth agenesis characterized by the absence of six or more permanent teeth. Oligodontia has complex etiology and variations in numerous genes have been suggested as causal for the condition.

**Methods:**

We applied whole‐exome sequencing (WES) to identify the cause of oligodontia in a 9‐year‐old girl missing 11 permanent teeth. Protein modeling and functional analysis in zebrafish were also performed to understand the impact of identified variants on the phenotype.

**Results:**

We identified a novel compound heterozygous missense mutation in *WNT10A* (c.637G>A:p.Gly213Ser and c.1070C>T:p.Thr357Ile) as the likely cause of autosomal recessive oligodontia in the child. Affected residues are located in conserved regions and variants are predicted to be highly deleterious for potentially destabilizing the protein fold and inhibiting normal protein function. Functional studies in zebrafish embryos showed that *wnt10a* is expressed in the craniofacies at critical time points for tooth development, and that perturbations of *wnt10a* expression impaired normal tooth development and arrested tooth development at 5 days postfertilization (dpf). Furthermore, mRNA expression levels of additional tooth development genes were directly correlated with *wnt10a* expression; expression of *msx1, dlx2b, eda,* and *axin2* was decreased upon *wnt10a* knockdown, and increased upon *wnt10a* overexpression.

**Conclusions:**

Our results reveal a novel compound heterozygous variant in *WNT10A* as pathogenic for oligodontia, and demonstrate that perturbations of *wnt10a* expression in zebrafish may directly and/or indirectly affect tooth development recapitulating the agenesis phenotype observed in humans.

## Introduction

Oligodontia is the severe form of tooth agenesis characterized by the developmental absence of six or more permanent teeth, not including the third molars (Hennekam et al. 2010). As the number of missing teeth increases, so does the severity of clinical consequences and the impact on oral health–related quality of life (Polder et al. 2004). In addition to esthetic implications that compromise an individual's confidence and self‐esteem, functional consequences of oligodontia include decreased masticatory function, malocclusion, and atrophy of subjacent maxillary or mandibular bone in the edentulous areas. Oral rehabilitation treatment for oligodontia requires extensive and costly procedures, especially when associated with additional dental anomalies and abnormal occlusion which further affect craniofacial development (Polder et al. 2004).

Oligodontia can occur in association with a variety of craniofacial syndromes, but most often is found as an isolated trait having an overall prevalence of ~1% in the general population. While isolated oligodontia often appears to have a sporadic occurrence, monogenic inheritance has been reported with autosomal dominant transmission being the most common. Autosomal recessive and X‐linked forms have also been observed (Hennekam et al. 2010). Despite considerable efforts to detect loci that contribute to isolated oligodontia susceptibility, gene identification has had limited success likely due to the genetic heterogeneity and variable expressivity of the condition, and the limitations of gene detection techniques (Yin and Bian 2015). The best characterized mutations involve muscle segment homeobox 1 *(MSX1,* OMIM 142983*)* (Vastardis et al. 1996) and paired box 9 (*PAX9,* OMIM 167416) (Stockton et al. 2000) genes, followed by ectodysplasin A (*EDA,* OMIM 300451) (Han et al. 2008)*,* axis inhibition protein (*AXIN2,* OMIM 604025) (Lammi et al. 2004), and more recently, Wnt family member 10A (*WNT10A,* OMIM 606268) (Bohring et al. 2009), LDL receptor‐related protein 6 (*LRP6,* OMIM 603507) (Massink et al. 2015), and Wnt family member 10B *(WNT10B,* OMIM 601906) (Yu et al. 2016), all of which have been implicated in oligodontia phenotypes. Furthermore, bioinformatics analyses of genes related to tooth agenesis have revealed additional gene pathways that may be involved in the etiology of the condition including those involved in tooth, skin, and gland development pathways, in addition to cancer pathways (Yin and Bian 2015).

Using whole‐exome sequencing, we recently identified novel and known variants in WNT pathway genes, and particularly in *WNT10A*, as pathogenic for familial oligodontia (Dinckan et al. 2017). In this study, we report the findings of a novel pathogenic compound heterozygous missense mutation in *WNT10A* in a patient with isolated oligodontia. Protein modeling and functional assays in zebrafish were performed and confirmed that perturbations in *wnt10a* expression resulted in impaired tooth development.

## Materials and Methods

### Ethical compliance

This study was approved by the UTHealth Committee for Protection of Human Subjects (HSC‐12‐0255). Written informed consent was obtained from all study participants and their legal guardians in the case of minors.

### Study subjects

Subjects were recruited at the UTHealth School of Dentistry clinics, based on clinical and radiographic examination findings showing the absence of one or more permanent teeth (excluding third molars), supporting the diagnosis of tooth agenesis. Peripheral blood samples were collected as source of genomic DNA. DNA extraction followed established protocols and the DNA concentration and quality were estimated using NanoDrop ND‐1000 (Nanodrop, Wilmington, DE).

**Figure 1:**
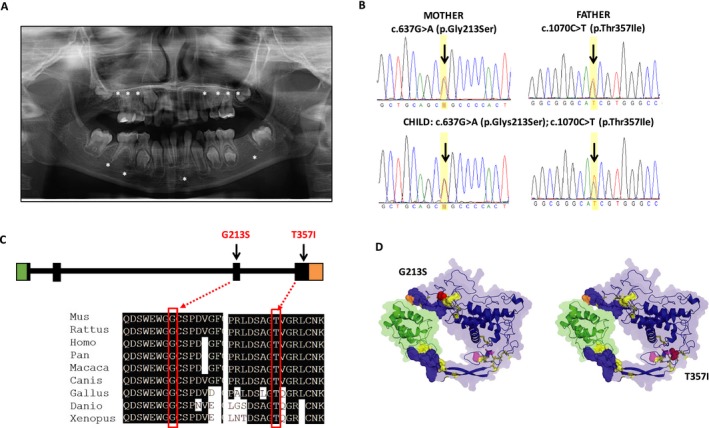
A compound heterozygous mutation in *WNT10A* [c.637G>A (p.Glys213Ser); c.1070C> (p.Thr357Ile)] causes isolated oligodontia. (A) Panoramic radiograph of proband with oligodontia. Radiograph shows a mixed dentition stage with the presence of deciduous teeth and some permanent teeth erupted. Stars denote absence of tooth buds for teeth # 3, 4, 5, 11, 12, 13, 14, 19, 24, 29, and 30. (B) Validation of the *WNT10A* mutations by Sanger sequencing. Each parent is a heterozygous carrier for one of the two mutations and the affected daughter is compound heterozygous for the c.637G>A (p.Glys213Ser) and c.1070C> (p.Thr357Ile) mutations (arrows in the sequencing chromatograms indicate the variant positions). (C) Schematic of *WNT10A* gene structure showing location of identified variants in exons 3 and 4, and conservation of glycine and threonine residues at positions 213 and 357 of the WNT10A protein, respectively. (D) Structural modeling analysis of WNT10A suggests both c.637G> A (p.Gly213Ser) and c.1070C> T (p.Thr357Ile) are highly deleterious and predicted to destabilize the protein fold and inhibit normal protein function. A homology model of WNT10A reveals a highly constrained fold with 11 disulfide bonds (yellow) and a two‐part binding site (refined from ModBase model 2, based on PDB 4F0A chain B with the binding partner from 4F0A shown in blue, based on structural alignment). The VIPUR pipeline predicts both G213S and T357I variants (red spheres) destabilize the protein fold and disrupt protein‐binding functions. The G213S variant is in a flexible region near four disulfide bonds and a palmitoyleyl modification site (orange). The change to serine at this position is predicted to disrupt the formation of these disulfide bonds, destabilizing this region, and preventing proper binding. This region also contains a palmitoylation site (orange) and destabilization of this region may prevent proper post‐translational modification of WNT10A. The T357I variant is located near three disulfide bonds in a very constrained region of the protein and a N‐acetylglucosamine modification site (magenta). Although not physically close to the binding site, this variant is predicted to disrupt the orientation of a “finger‐like” projection at the binding site, eliminating the proper arrangement of the binding regions.

The discovery family consisted of a nuclear family of a 9‐year‐old Asian girl with oligodontia and her parents. Examination of the child revealed absence of 11 permanent teeth (# 3, 4, 5, 11, 12, 13, 14, 19, 24, 29, 30) (Fig. 1A). The child's primary dentition was normal, and both parents had all 32 permanent teeth. No evidence of syndromes or structural abnormalities was found in the child or parents, and family history of missing teeth was negative. Similarly, no defects in tooth shape, hair, skin, or nails were noted in the child or unaffected parents.

### Whole‐exome sequencing

Whole‐exome sequencing was performed on DNA samples of the child proband (II.1) after Sanger sequencing excluded mutations in *MSX1, PAX9,* and *AXIN2* genes, commonly associated with oligodontia. Exome capture was performed using the Agilent SureSelect Human All Exon 50 MB kit (Agilent Technologies, Santa Clara, CA) followed by massively parallel sequencing on an Ilumina HiSeq 2000 platform (Illumina, San Diego, CA). Paired‐end reads were aligned against the human reference genome (UCSC Human Genome Browser hg19) using the Burrows‐Wheeler Aligner (BWA, v.0.6.1) (Li and Durbin 2009). PCR duplicate removal, indel realignment, and variant calling were performed using the Genome Analysis Toolkit (GATK, v.1.4) (McKenna et al. 2010).

The dbNSFP database (Liu et al. 2016) and ANNOVAR (Wang et al. 2010) were used to annotate variants as frame shift, stop‐gain, stop‐loss, disruption of splice sites, missense, synonymous, UTR, or noncoding. Variants were filtered to exclude: (1) variants with a minor allele frequency >0.01 in dbSNP, in the NHLBI Exome Variant Server (www.evs.gs.washington.edu), or in the 1000 Genomes Project Database (www.1000genomes.org), (2) synonymous variants, and (3) intronic variants >5 bp from exon boundaries. Variants were classified as deleterious (potentially pathogenic), benign, or of unknown clinical significance, by the algorithms SIFT, PolyPhen‐2, Mutation Taster, Mutation Assessor (implemented in the dbNSFP database), and VIPUR (Baugh et al. 2016). Deleterious mutations and variants of unknown clinical significance were classified as related or unrelated to the phenotype, and as recessive mutations, or mutations with no known disease associations, using the American College of Medical Genetics and Genomics (ACMG) guidelines. Additional filtering on the basis of gene function and expression of individual genes during craniofacial and/or dental development was also performed using the aforementioned databases. Sanger sequencing was used to confirm potentially pathogenic variants identified. Sequence alterations were reported according to the Human Genome Variation Society (HGVS) nomenclature guidelines.

### Variant validation

To further determine the frequency of the *WNT10A* variants in tooth agenesis, variants identified by whole‐exome sequencing and eight additional known variants in the *WNT10A* gene were investigated using custom‐made Taqman SNP genotyping assays (Applied Biosystems, Foster City, CA) (Table S1) in an additional case–control dataset composed of 97 unrelated self‐reported Caucasian individuals with isolated tooth agenesis and 360 controls. No syndromic features were identified in any individuals.

### Molecular modeling

Sequence homology of human *WNT10A* (NM_025216.2) to other species was assessed using ClustralW2 (http://www.ebi.ac.uk/Tools/clustalw2/index.html). Protein sequence accession numbers were as follows: mouse (Mus), NP_033544.1; rat (Rattus), NP_001101697.1; human (Homo), NP_079492.2; chimp (Pan), XP_516098.2; monkey (Macaca), XP_001095740.1; wolf (Canis), XP_545648.2; chicken (Gallus), NP_001006590.1; zebrafish, (Danio) NP_571055.1; and frog (Xenopus), XP_002934004.2 (Fig. S1). Assessment of potential consequences and interpretation of deleterious variants were performed using VIPUR (Baugh et al. 2016), with a WNT10A homology model based on PDB 4F0A chain B (*Xenopus* WNT8) and 37% sequence identity to the template structure.

### Zebrafish functional assay

#### Zebrafish husbandry and care

Zebrafish (*Danio rerio*) wild‐type Dz and AB strains were maintained at 28.5°C, and embryos were collected and staged according to established methods (Westerfield 1995; Sprague et al. 2006). All work involving animals was performed in accordance with the Institutional Animal Care and Use Committee (IACUC) at the University of Texas Health Science Center at Houston, the Rice University Institutional Animal Care and Use Committee guidelines. Experiments were repeated three times and a Pearson's chi‐square test was used to determine the significance.

#### Morpholino injections

Reciprocal BLAST was used against the *Danio rerio* genome and identified a sole zebrafish ortholog of *WNT10A* (ENSDARG00000017155, 74% identity). Two zebrafish wnt10a antisense morpholinos (MO) and one mismatch control MO were designed by GeneTools (Philomath, OR): (1) translation blocking MO (tbMO) targeting the ATG site in exon 1 (GTCGTGAGAGCTCATTCATGGAATC); (2) splice‐blocking MO (sbMO) targeting the exon2/intron2 junction (CTGTTTGATTTGATCGCTTACCCCT); and (3) mismatch control MO (GTCaTaAGAaCTCATTgATaGAATC) (Fig. S2). All MOs were diluted in nuclease‐free water to a stock concentration of 65 mg/mL or 2 mmol/L. Injection MOs (2.0 mg/mL) were diluted in Danieu buffer (58 mmol/L NaCl, 0.7 mmol/L KCl, 0.4 mmol/L MgSO_4_, 0.6 mmol/L Ca(NO_3_)_2_, 5.0 mmol/L HEPES pH 7.6) and 0.5 uL of 2% phenol red was added to facilitate injections. One‐cell embryos were injected with 1 nL of MO and observed during development up to 8 days postfertilization (dpf). Injection volume was calculated by measuring the diameter of injected droplet. Embryos were incubated at 28°C and collected at appropriate time points.

#### Whole‐mount in situ hybridization

An antisense RNA probe for *wnt10a* was made by insertion of a reverse T7 promoter in pCS2 + Wnt10a plasmid using the primers (Fwd: CAAGCTTGATTTAGGTGACAC, Rev: GCATTTTTTTAATACGACTCACTATAGGGTCACTGCATTCTAGTTGTGG). Probe was transcribed using digoxigenin‐11‐UTP (Roche, Indianapolis, IN) and T7 polymerase (Promega, Madison, WI). Whole‐mount in situ hybridization was carried out as described (Sprague et al. 2006).

#### Alcian blue and alizarin red staining

In order to visualize cartilage and bone structures during zebrafish embryo development, alcian blue (Anatech LTD, Battle Creek, MI) and alizarin red (Sigma‐Aldrich, St. Louis, MO) staining was performed using standard techniques (Kimmel and Trammell 1981). Briefly, morpholino‐injected and uninjected control (UIC) embryos from 3 to 8 dpf were collected and fixed in 2% paraformaldehyde/1X PBT for 1 h at room temperature and stored in methanol over night at −20°C. After removing methanol, embryos were incubated in 0.04% alcian blue solution (100 mmol/L Tris, pH 7.5, 10 mmol/L MgCl_2_, 64% ethanol). Following destaining in 3% H_2_O_2_/0.5% KOH for 10 min at room temperature, embryos were stained in 0.02% alizarin red solution containing 100 mmol/L Tris, pH 7.5, 25% glycerol for 30 min at room temperature. Embryos were destained in 50% glycerol/0.1% KOH for 30 min and stored in 50% glycerol. Imaging was performed using the LAS Montage Module (Leica, Wetzlar, Germany).

#### Western blot

Cell lysates were prepared from 4 hpf and 24 hpf UIC, *wnt10a* Tb‐ and SbMO‐injected embryos, and *wnt10a* RNA‐injected embryos, and dechorionated with pronase. After de‐yolking manually on an agarose plate, the embryos were collected and mixed with SDS sample buffer and boiled for 10 min. Proteins were resolved by SDS‐PAGE electrophoresis (4–12% gradient, NuPAGE Novex Bis‐Tris Gel 1.0 mm) (Life Technologies, Foster City, CA) and then transferred onto PVDF membrane (Invitrogen iBlot Dry, Carlsbad, CA). The membrane was incubated with primary antibodies anti‐wnt10a (cat. ARP41277_P050, LI‐COR Biosciences, Lincoln, NE) and antiactin (cat. Ab8227, Abcam, Cambridge, UK) at room temperature for 2 h. Following washing three times with 1xTBST for 15 min each, the membrane was incubated with IRDye^**®**^ 800 CW antirabbit IgG secondary antibody (cat. 926‐32211, LI‐COR) for 1 h at room temperature. Protein bands were visualized using LI‐COR Odyssey Scanning Imager (LI‐COR).

#### Quantitative RT‐PCR

*wnt10a* TbMO‐injected embryos, *wnt10a* mismatch control (MM)‐MO‐injected embryos, and *wnt10a* RNA‐injected embryos were collected at different time points (18hpf, 24hpf, 2dpf, 3dpf, 4dpf, and 5dpf) and homogenized in Trizol reagent (Invitrogen). Total RNA was isolated using RNeasy Mini kit (Qiagen, Valencia, CA), and treated with DNase I. Complementary DNAs (cDNA) were synthesized from 1 µg of total RNA using the iScript cDNA synthesis kit (Bio‐Rad, Hercules, CA) according to manufacturer's instructions. Expression levels of *wnt10a* (ENSDARG00000017155), *msx1* (ENSDARG0000000 7641), *eda* (ENSDARG00000074591)*, dlx2b* (ENSDARG 00000017174), and *axin2* (ENSDARG00000100149) mRNA were evaluated by qRT‐PCR using SYBR^®^ Green PCR Master Mix (Applied Biosystems) in a Viia7 sequence detection instrument (Applied Biosystems). Assays were conducted in triplicates. Quantitative expression values were extrapolated from separate standard curves, and normalized to *β*‐actin as internal control. Fold‐changes were calculated by the 2^−∆∆Ct^ method (Schmittgen and Livak 2008) and analyzed using Student's *t* test. *P*‐values ≤ 0.05 were considered statistically significant. Primers for *wnt10a, dlx2b, msx1, axin2, eda,* and *beta‐actin* were commercially available or designed using Primer 3 (Rozen and Skaletsky 2000) (Table S2). The specificity of the primer sets was confirmed by the presence of a single peak in the dissociation curve analysis and by detection of a single band of the correct size by gel electrophoresis.

#### Molecular methods and microinjection

Total mRNA was isolated from zebrafish embryos collected at 24‐h postfertilization (hpf). Total mRNA (1 µg) was used as template to generate a cDNA library using Bio‐Rad iScript cDNA synthesis Kit (Bio‐Rad). Zebrafish full‐length wnt10a was amplified from the cDNA library using primers with a EcoRI restriction enzyme (NEB, Ipswich, MA) cut site added at both ends for cloning purposes (Fwd: AAGGTTCCGAATTC***ATG**AG* ***T*** *TC* ***C*** *CA* ***T*** *GA* ***T*** *ATC*AGTTGGCACTCTCCAGC and Rev: AAGGTTCCGAATTC**TCA**TTTGCAGACACTGACCCAC). The Forward (Fwd) primer contains partial morpholino target site (italicized). Four‐point synonymous mutations (underlined) were introduced in the morpholino target site to create mismatches to the morpholino in order to prevent morpholino binding. PCR products digested with EcoRI were then cloned using T4 DNA ligase into pCS2 vector, which had been digested with EcoRI and treated with alkaline phosphatase (Sigma) prior to ligation. One Shot TOP10 competent cells (Invitrogen) were used for transformation of the ligation reaction. pCS2‐wnt10a plasmid DNA was prepared using Qiagen plasmid mini kit (Qiagen). Clones with correct wnt10a full‐length sequence (NM_130980) were identified through DNA sequencing. Human full‐length WNT10A‐expressing vector pCS2‐WNT10a (NM_025216) was purchased from GeneScript.

#### Overexpression of *Danio rerio* and *Homo sapiens* Wnt10a in zebrafish

Mutated versions of *Danio rerio* (Dr) *wnt10a* and *Homo sapiens* (Hs) *WNT10A* were created using the QuikChange XL Site‐Directed Mutagenesis Kit (Stratagene, San Diego, CA). Primer sequences are available in Table S4. In vitro transcribed RNA was synthesized using the mMESSAGE mMACHINE kit (Ambion, Carlsbad, CA). For microinjection, Dr wnt10a and Hs mRNAs were injected into the yolk of zebrafish embryos at one‐ or two‐cell stage. mRNAs were diluted in 0.1 mol/L KCl to 1.0 *μ*g/mL and injected 1 nL/embryo.

## Results

### Identification of a compound heterozygous variant in WNT10A

Whole‐exome sequencing was performed in the proband presenting with oligodontia and absence of 11 permanent teeth (Fig. 1A). WES mean target coverage was 64x, and the percentage of the WES target covered by at least 10 reads was 92%. Over ~155,000 variants were identified in the child with 37,748 variants identified on target regions. Upon filtering, 3132 variants that were not in dbSNP were further reduced to 521 with MAF<0.01. Additional filtering was performed to prioritize variants on the basis of gene function and expression of individual genes during craniofacial and/or dental development. Finally, filtering for deleterious variants under an autosomal‐recessive model revealed two heterozygous missense variants in *WNT10A* as potentially pathogenic: c.637G>A (p.Gly213Ser) and c.1070C>T (p.Thr357Ile), located in exons 3 and 4 of the *WNT10A* gene, respectively. Sanger sequencing of exon/intron boundaries confirmed the presence of both variants in the affected child, whereas unaffected parents were found to be carriers for each variant (c.1070C>T in the father and c.637G>A in the mother) (Fig. 1B). The c.1070C>T (p.Thr357Ile) variant was not found annotated in any of the genetic variation databases, although it has been reported in one Chinese individual with isolated oligodontia (of 500 cases) (16). The c.637G>A (p.Gly213Ser) mutation is very rare in most populations, although a frequency of 0.01 in Asians is reported in the 1000 Genomes Project Database.

### Predicted impact of WNT10A variants

The individual variants c.637G>A (p.Gly213Ser) and c.1070C>T (p.Thr357Ile) affect highly conserved amino acids and are predicted as deleterious (Fig. 1C). While no crystal structure of the human WNT10A protein is currently available, functional annotation of a WNT10A homology model suggests that both variants introduce larger amino acids that destabilize nearby disulfide bonds (Fig. 1D). Wnt proteins are highly conserved in vertebrates (Fig. S1) and have a remarkably high number of disulfide bonds. The c.637G>A (p.Gly213Ser) variant is located in a coiled coil domain (two positions away from the helix domain) with four nearby disulfide bonds (C159–C214, C149–C157, C262–C276, and C264–C271). The c.1070C>T (p.Thr357Ile) variant is in a highly constrained region with three disulfide bonds (C346–C377, C376–C416, and C362–C372) that properly orient a “finger‐like” projection that forms part of the proteins binding site and is also in close proximity to a N‐glycosylation site located between residues 363 and 366 (data not shown). Using the VIPUR pipeline, the c.637G>A (p.Gly213Ser) variant (0.91 score) was predicted to destabilize the entire domain around it by preventing proper formation of disulfide bonds, which in turn may inhibit palmitoylation of residue 268, a known palmitoyl modification site contained within this same domain (Fig. 1D, position 268 is colored orange). Similarly, c.1070C>T (p.Thr357Ile) (0.80 score) is predicted to destabilize the disulfide bonds that orient part of the predicted binding site and may inhibit carbohydrate modification of nearby residues (Fig. 1D, carbohydrate modification sites are colored magenta). This structural analysis suggests that both alleles have significantly reduced or completely impaired ability to interact with the receptor(s) of WNT10A as proper orientation of both binding regions is likely required for sufficient binding. While the destabilization predicted for these variants appears to disrupt WNT10A‐binding interactions, they may also disrupt interactions with enzymes performing posttranslational modifications to WNT10A and contribute to improper localization (inhibited secretion in this case). Both variant scores are comparable with other disease‐associated variants in *WNT10A* including p.Trp277Cys (0.99 score), p.Arg360Cys (0.96 score), and p.Phe228Ile (0.91 score) (Table S1), and are nearly as disruptive as the predicted deleterious impact of eliminating disulfide bonds.

### Additional WNT10A variants found in unrelated cases

The known heterozygous missense variant c.682T>A (p.Phe228Ile) was identified in one patient with oligodontia of the 97 cases with tooth agenesis. None of the tested variants were found in the unaffected individuals.

### Perturbation of wnt10a expression impairs tooth development in zebrafish embryos

**Figure 2:**
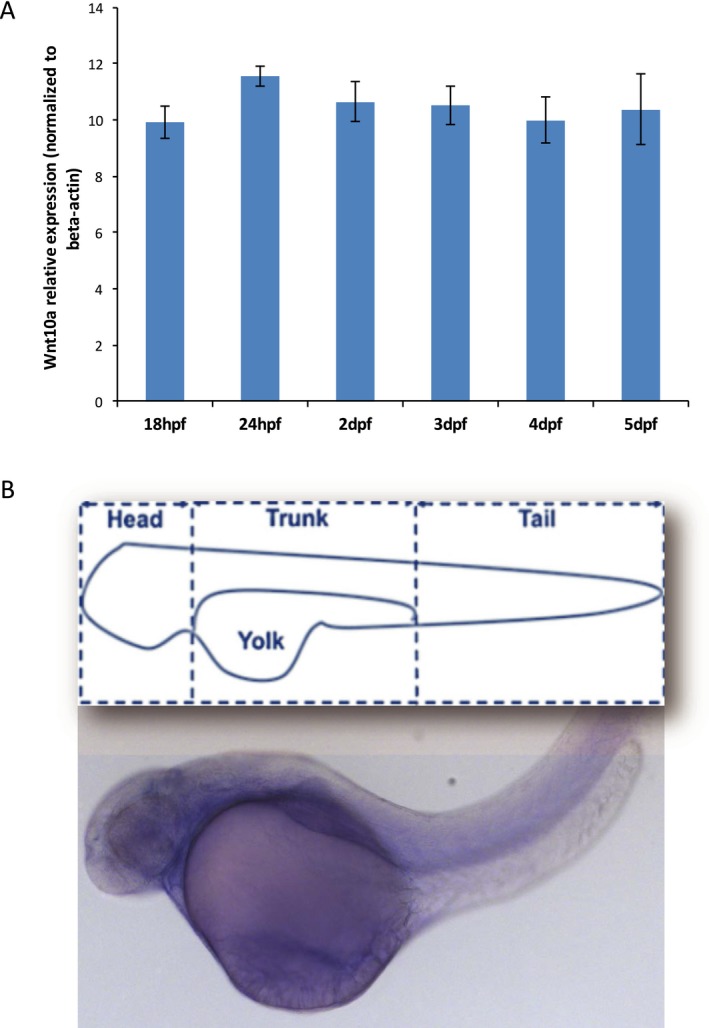
*wnt10a* is expressed during zebrafish embryonic development. (A) *wnt10a* mRNA expression was detected by qPCR analysis in zebrafish craniofacies at critical time points for tooth development (18hpf, 24hpf, 2dpf, 3dpf, 4dpf, and 5dpf). (B) Schematic of zebrafish structure and whole‐mount in situ hybridization of zebrafish embryos at 56hpf showing widespread and uniform expression of *wnt10a* mRNA.

Zebrafish was used to gain insights into the role of *wnt10a* during craniofacial and tooth development. We first assessed its expression in wild‐type zebrafish embryo heads by real‐time PCR. *wnt10a* expression was detected throughout developmental stages preceding and following critical time points for tooth development (18hpf, 24hpf, 2dpf, 3dpf, 4dpf, and 5dpf), although no significant differences were observed between each stage (Fig. 2A). Whole mount in situ hybridization of zebrafish embryos at 56hpf showed widespread and uniform expression (Fig. 2B).

**Figure 3:**
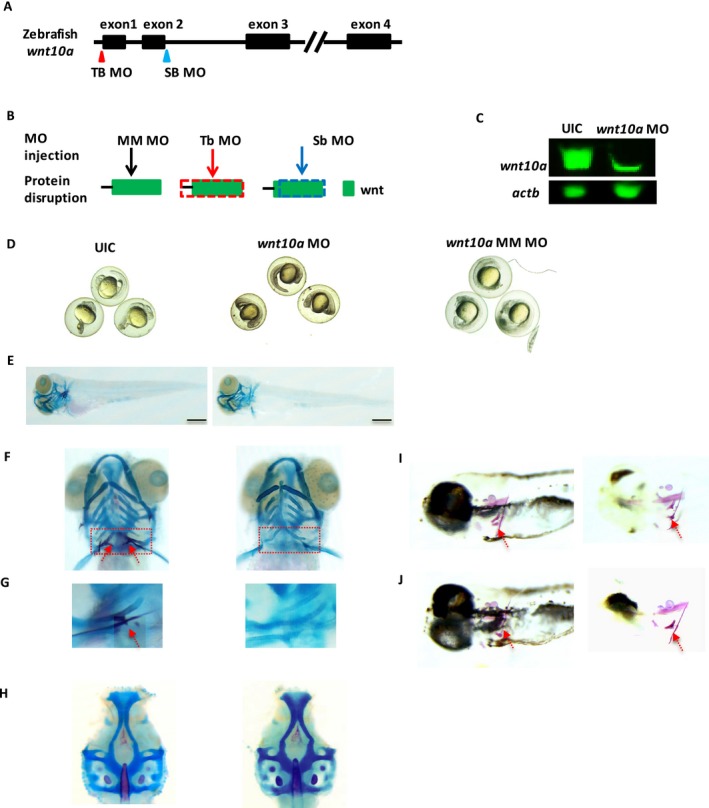
Knockdown of *wnt10a* expression impairs tooth development at 5dpf and recapitulates human tooth agenesis phenotype. (A) Morpholino knockdown of zebrafish *wnt10a*. Target sites for translation‐blocking (TBMO, red triangle) and splice site‐blocking (SBMO, blue triangle) morpholinos. (B) Morpholinos were used to block the translation initiation complex (TB) or the locus involved in splicing pre‐mRNA (SB), or used as mismatch control (MM). Wnt10a protein production is disrupted at dotted frames for TB (red frame) and SB (blue frame). (C) Western blot analysis confirming knockdown efficiency. Wnt10a level was largely reduced in the MO‐injected zebrafish embryos compared to uninjected control (UIC) embryos. Beta‐actin was used as loading control. (D) UIC,* wnt10a*‐MO‐injected fish and mismatch‐MO‐injected embryos at 24hpf do not present gross body abnormalities. (E–H) Alcian blue and alizarin red staining of: (E) UIC,* wnt10a* morpholino‐injected (MO) zebrafish at 5dpf showing similar body structure and appearance, and (F,G) the presence of pharyngeal teeth in UIC (red arrow, left panel), but not in wnt10a‐MO‐injected embryos (right panel). (G) Higher magnification of dotted box in (F). (H) UIC control zebrafish embryos had no noticeable cartilage defects, whereas wnt10a‐MO‐injected fish presented with some cartilage abnormalities**.** (I, J) Mismatch morpholino‐injected embryos at 5dpf develop normally and present with pharyngeal teeth (red arrows) similarly to UIC embryos (embryos were cleared to enhance mineralized structures). Scale bar represents 150 µm.

Next, to evaluate the potential effects of *wnt10a* loss of function on zebrafish tooth development, zebrafish at one‐cell stage were injected with either a translation blocking (tbMO) or splice‐blocking (sbMO) morpholino (Fig. 3), and observed up to 8 days postfertilization (dpf). Uninjected and mismatch MO‐injected embryos were used for comparison. At 24 h postfertilization (hpf), similar survival rates were observed for both tbMO‐ and sbMO‐injected embryos and uninjected controls (Fig. S2). Thereafter, survival rates decreased in the MO‐injected embryos albeit not significantly different from controls. While neither tbMO‐ nor sbMO‐injected embryos had gross whole‐body abnormalities, some defects in cartilage formation were observed (Figs. 3, S3), and tooth development was arrested in 94–97% of the fish by 5 dpf, recapitulating the tooth agenesis phenotype observed in humans (Fig. 3, Table S3). In contrast, 100% of uninjected control fish presented with normal teeth. Mismatch control–morpholino‐injected embryos did not present any phenotype and were identical to uninjected control embryos (Fig. 3), indicating that off‐target effects were unlikely. These findings indicate that *wnt10a* knockdown arrests or severely impairs zebrafish tooth development.

**Figure 4:**
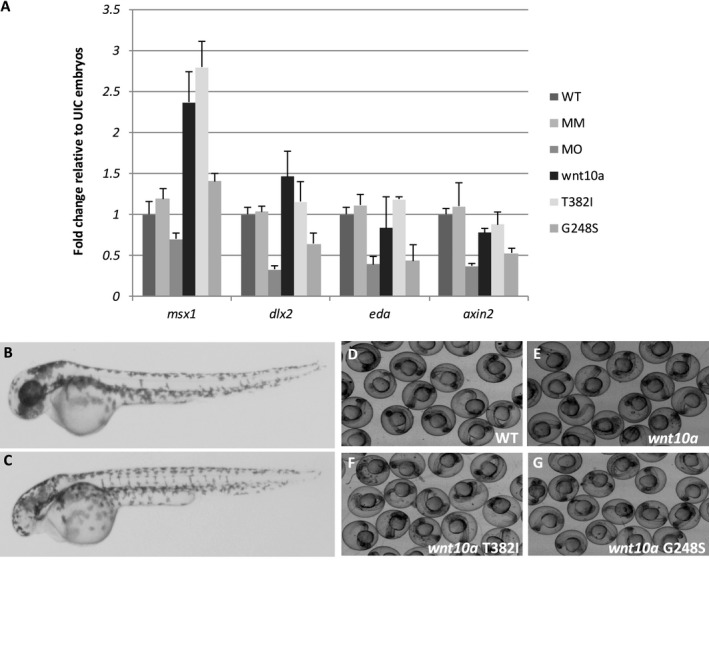
Perturbation of *wnt10a* expression results in altered expression of additional tooth development genes at tooth bud formation stage (2 dpf). (A) Quantitative RT‐PCR analyses of *msx1*,* dlx2b, eda,* and *axin2* genes in WT, mismatch‐MO injected (MM), *wnt10a*‐MO injected (MO), *wnt10a* RNA injected (wnt10a), and *wnt10a* mutant RNA with T328I and G248S mutations (corresponding to T357I and G213S in humans). No differences were observed in the expression of those genes between WT and MM‐injected embryos, whereas knockdown of *wnt10a* led to significantly decreased expression of *dlx2b, eda,* and *axin2* (*P* = 0.001). In contrast, overexpression of wnt10a resulted in increased expression of all four genes (*P* < 0.02), although with significant differences only for *msx1* (*P* = 0.01) and *axin2* (*P* = 0.02). Injection of mutant wnt10a RNAs also perturbed expression of *msx1*,* dlx2b, eda,* and *axin2*, particularly the G248S mutant (0.002 < *P* < 0.02). Target gene expression was normalized to the expression of beta‐actin used as endogenous control. Fold changes were calculated in relation to controls by the 2^−∆∆Ct^ method and analyzed using Student's *t* test. *P*‐values ≤0.05 were considered statistically significant. (B,C) Overexpression of Wnt10a leads to a small/no eyes phenotype in wnt10a RNA‐injected fish (C) in comparison to WT (B) at 2 dpf. Embryos not injected with any RNA (D), injected with 1 pg zebrafish *wnt10a* RNA (E), 1 pg *wnt10a* RNA with T382I mutation (F), and 1 pg *wnt10a* RNA with G248S mutation (G).

To determine if perturbations in *wnt10a* expression would affect the expression of additional tooth development genes, we evaluated the expression of *msx1, dlx2b, eda,* and *axin2* in wild type, *wnt10a* tbMO‐injected fish, mismatch MO‐injected fish, as well as wnt10a RNA‐injected, and injected with wnt10a T382I and G248S mutant alleles (which corresponds to the human alleles). At 2 dpf, a significant reduction in the expression of *dlx2b, eda,* and *axin2* (*P* = 0.001) was observed in *wnt10a* MO‐injected embryos. In contrast, overexpression of wnt10a resulted in increased expression of all four genes, with significant differences observed for *msx1* (*P* = 0.01) and *axin2* (*P* = 0.02). Injection of mRNA encoding the *wnt10a* mutant alleles also perturbed expression of *msx1*,* dlx2b, eda,* and *axin2*, particularly the G248S mutant (0.002 < *P* < 0.02) (Fig. 4A). We also confirmed that the ubiquitous expression of wild‐type human or zebrafish *wnt10a* resulted in loss of anterior neural identity causing embryos to show a small/no eyes phenotype (Fig. 4B), as seen for overexpression of other canonical Wnt genes (Kelly et al. 1995; Lekven et al. 2001). A dose‐sensitive loss of anterior neural structures was also observed for wild‐type and mutant mRNAs encoding the Gly213Ser or Thr357Ile mutations (Fig. 4B‐4G).

## Discussion

Numerous studies have implicated a major role for *WNT10A* in the etiology of tooth agenesis in humans (van den Boogaard et al. 2012), despite the opposing supernumerary teeth phenotype observed in *Wnt10a* null mice (Yang et al. 2015; Xu et al. 2017). Here, we report the clinical and functional impact of a compound heterozygous mutation in *WNT10A* (c.637G>A (p.Gly213Ser); c.1070C>T (p.Thr357Ile) as likely pathogenic for isolated oligodontia, and demonstrate that perturbations in *wnt10a* expression impairs normal tooth development in zebrafish embryos.

The *WNT10A* gene is a member of the WNT gene family, which consists of a large family of secreted glycoproteins that specify various cell lineages during embryogenesis (Liu and Millar 2010). During tooth development, continuous Wnt‐*β*‐catenin signaling in the dental epithelium and mesenchyme is required for tooth formation and morphogenesis (Jarvinen et al. 2006; Liu and Millar 2010). *WNT10A* is thought to function through the canonical Wnt‐*β*‐catenin signaling pathway and activates target genes in the nucleus through *Lef1* transcriptional factor activity (Eastman and Grosschedl 1999).

The individual mutations (c.637G>A [p.Gly213Ser]; c.1070C>T [p.Thr357Ile]) identified here are rare and predicted to affect highly conserved amino acids with deleterious effects to the WNT10A protein structure and function. Furthermore, each mutation is predicted to be individually disruptive of protein function with potential additive effects. For example, these variants are likely destabilizing, or may disrupt post‐translational modifications and inhibit proper localization, or eliminate disulfide bonds, which are sufficient to cause loss of molecular function. Wnt proteins are highly conserved in vertebrates and have a remarkably high number of disulfide bonds, suggesting that protein conformation is important for correct interaction with their receptors. This is important because WNT10A likely requires the proper orientation of both binding regions to interact with its receptor(s) (Lekven et al. 2001).

Compound homozygous and heterozygous, and single heterozygous mutations in *WNT10A* have been suggested to contribute to up to 50% of the tooth agenesis cases (van den Boogaard et al. 2012; Dinckan et al. 2017). However, interpretation of each *WNT10A* variant's contribution to tooth agenesis phenotypes remains limited by the lack of information about which variant alleles may be contributing to the phenotype. In general, *WNT10A* compound heterozygous mutations have been found in association with oligodontia and a greater number of missing teeth when compared to individuals with a single mutation (Kantaputra and Sripathomsawat 2011; van den Boogaard et al. 2012; Arzoo et al. 2014; Bonds et al. 2014; Song et al. 2014; Vink et al. 2014). These compound heterozygous mutations were also more frequently associated with the absence of maxillary and mandibular molars as well as mandibular central incisors (Vink et al. 2014). Meanwhile, carriers of a single heterozygous *WNT10A* variant may not have tooth agenesis (Arzoo et al. 2014). Overall, these observations reflect our results, in which the child with the compound heterozygous *WNT10A* mutation has oligodontia and each unaffected parent is a carrier for each individual mutation. In our confirmation case–control dataset, only one case presented with a known *WNT10A* variant (c. 682T>A; p.Phe228Ile), while no *WNT10A* variant was found in unaffected individuals.

Similarly, interpretation of the role of *Wnt10a* in a mouse model remains questionable and fails to elucidate the role of this gene in tooth agenesis. Evidence supporting a role for Wnt10a in the developing mouse tooth shows its expression in the enamel knot, and then shifting from the secondary enamel knot to the underlying mesenchyme to be continuously expressed in the developing odontoblasts (Yamashiro et al. 2007; Chen et al. 2009). Expression of *Wnt10a* induced and colocalized with mRNA expression of *Dspp* (a key molecule for dentin mineralization) in fully differentiated secretory odontoblasts, indicating that *Wnt10a* is potentially involved in dentin mineralization events as an upstream regulator of *Dspp* (Yamashiro et al. 2007). Interestingly, however, *Wnt10a*‐null and ‐mutant mice show supernumerary teeth (instead of missing teeth) and molar teeth characterized by taurodontism and altered molar crown morphology (Yang et al. 2015; Xu et al. 2017). These findings are opposite to the phenotypes consistently reported for individuals with *WNT10A* variants in all reported human genetic studies.

To gain better insights into the role of *WNT10A* during craniofacial and tooth development, we performed functional assays in zebrafish embryos. Zebrafish have pharyngeal teeth that are located on the fifth ceratobranchials, and are replaced continuously throughout life, indicating that zebrafish teeth are likely to have a different germ layer origin of their epithelium than the teeth of the mouse but do have a conserved requirement for FGF signaling (Huysseune et al. 1998; Jackman and Stock 2006). The first tooth starts to develop around 48 hpf and is replaced at the same position at around 80 hpf; meanwhile the ventral row is completed at around 16 dpf. At the age of ~26 dpf, the zebrafish dentition is fully established and teeth are replaced throughout the life of the fish with each tooth being replaced every 8–12 days (Van der Heyden and Huysseune 2000). Our expression analysis confirmed the expression of *wnt10a* during critical time points for tooth development (18hpf, 24hpf, 2dpf, 3dpf, 4dpf, and 5dpf), implicating a role for *wnt10a* during tooth development in zebrafish embryos.

As zebrafish teeth are continuously replaced throughout life, we focused our analyses to reflect development of the first tooth, usually by 4–5 dpf, and our findings indicate that *wnt10a* knockdown arrests or severely impairs tooth development in almost all of the embryos with no additional gross structural abnormalities. Furthermore, perturbations of *wnt10a* expression resulted in altered expression of additional tooth development genes such as *msx1, dlx2b, eda,* and *axin2* (Borday‐Birraux et al. 2006; Zeng et al. 2008). Knockdown of *wnt10a* expression caused a significant reduction in the expression of *msx1, dlx2b, eda,* and *axin2*, thus suggesting that imbalances in *wnt10a* expression may directly or indirectly contribute to arrest or impairment of tooth development in zebrafish. In contrast, overexpression of *wnt10a* resulted in an increase in the expression of *msx1, dlx2b, eda,* and *axin2*. Moreover, in our overexpression assays of wild‐type human or zebrafish Wnt10a, we found loss of anterior neural identity in a dose‐dependent manner similar to that reported for overexpression of other canonical Wnt genes (Lekven et al. 2001). We also observed that the mutant mRNAs had similar potency as wild type indicating that these missense mutations retained some activity. This is likely due to the fact that *wnt10a* is acting outside of its normal signaling context and that mutations of these conserved residues affect specific regulation of *wnt10a* signaling in tooth formation, perhaps reflecting regulation of diffusion or coreceptor interaction. As the development of zebrafish teeth is relatively late, MO injections are unlikely to eliminate *wnt10a* expression completely, and some gene activity is still expected.

In conclusion, our findings provide evidence that *WNT10A* is critical for tooth development and mutations in this gene lead to arrest of normal tooth development in humans and in zebrafish. Moreover, we demonstrate that perturbation in *wnt10a* expression leads to altered expression of additional tooth development genes suggesting that *wnt10a* may have direct and indirect roles in tooth development.

## Conflict of Interest

The authors have no conflicts of interest to declare.

## Supporting information


**Figure S1.** WNT10A amino acid alignment across vertebrate species.
**Figure S2.** Analysis of zebrafish *wnt10a* morphant embryos.
**Figure S3.** *wnt10a* knockdown causes cartilage abnormalities in zebrafish.
**Table S1.** *WNT10A* custom‐SNPs used for genotyping.
**Table S2.** Morpholino and primer sequences used in this study.
**Table S3.** Number of embryos with and without teeth.
**Table S4.** PCR primers used for *wnt10a* overexpression and analysis of mutant alleles.
