# Supplementary material for: Role of WNT10A in failure of tooth development in humans and zebrafish
Source: Mol Genet Genomic Med. 2017 Sep 14;5(6):730–41. doi: 10.1002/mgg3.332 (PMC5702573; doi:10.1002/mgg3.332)
Supplement: Supplementary file 1 — Figure S1. WNT10A amino acid alignment across vertebrate species. Figure S2. Analysis of zebrafish wnt10a morphant embryos. Figure S3. wnt10a knockdown causes cartilage abnormalities in zebrafish. Table S1. WNT10A custom‐SNPs used for genotyping. Table S2. Morpholino and primer sequences used in this study. Table S3. Number of embryos with and without teeth. Table S4. PCR primers used for wnt10a overexpression and analysis of mutant alleles. [file MGG3-5-730-s001.docx]

**SUPPLEMENTARY MATERIAL**

**Role of *WNT10A* in failure of tooth development in humans and zebrafish**

Q. Yuan, M. Zhao, B. Tandon, X. Liu, L. Maili, A. Zhang, E.H. Baugh, T. Tran, R. Silva, J.T. Hecht, E.C. Swindell, D.S. Wagner, A. Letra


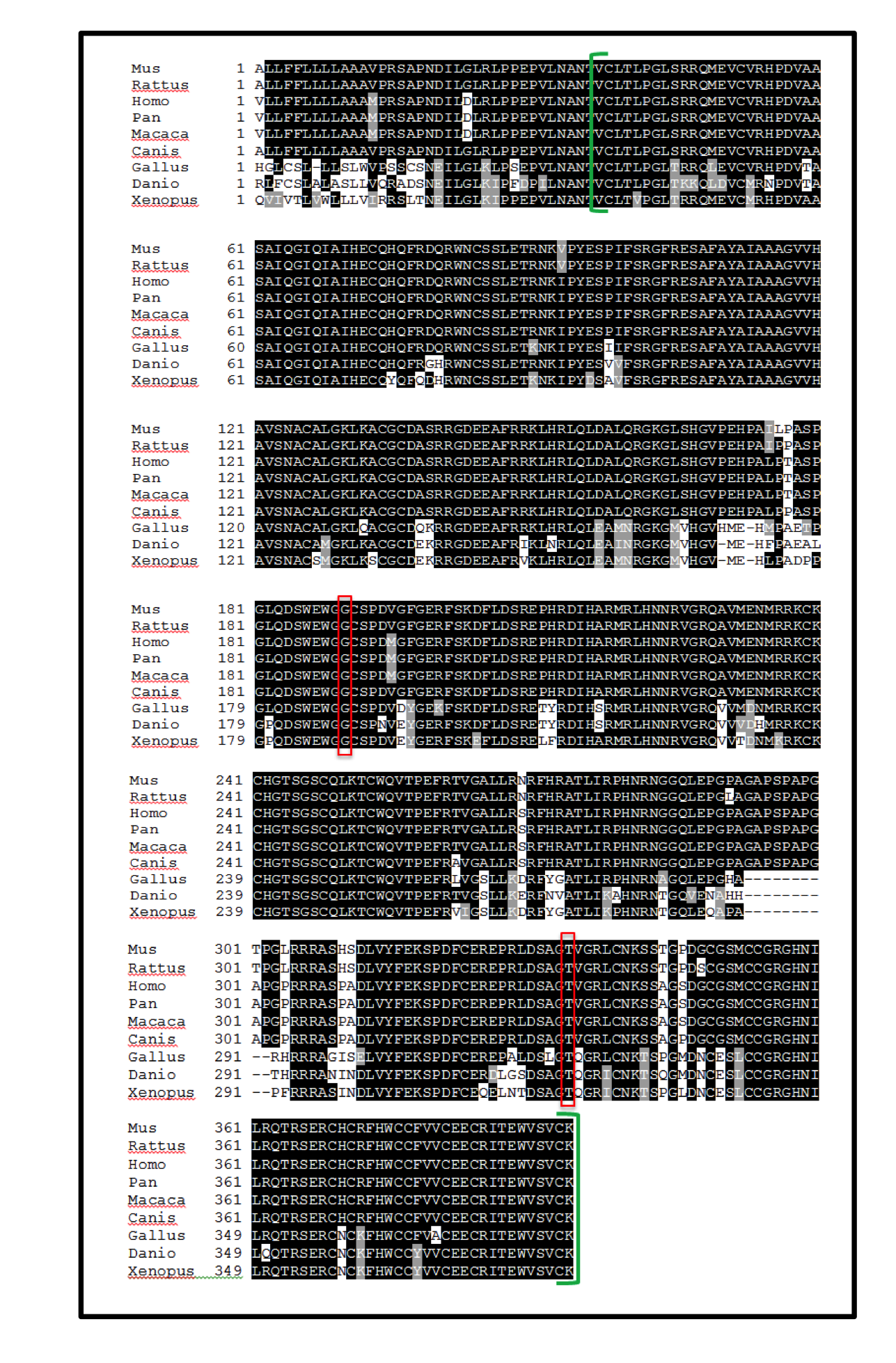


**Supplementary Fig. 1. WNT10A Amino Acid Alignment Across Vertebrate Species.** Multiple Sequence Alignment was performed by Clustal Omega and Boxshade. The *wnt10a* gene is conserved across species and has 76% identity at the amino acid level between human and zebrafish. The sequence of conserved domain wnt (accession pfm00110) is indicated in [ ]. The locations of the mutated residues G213S and T357I are highlighted with [] and demonstrate the strong conservation of both amino acids. Black boxes indicate >50% of sequences have the identical amino acid; grey boxes indicate >50% of sequences have a conserved substitution. Protein sequence accession numbers are: mouse (Mus), NP_033544.1; rat (Rattus), NP_001101697.1; human (Homo), NP_079492.2; chimp (Pan), XP_516098.2; monkey (Macaca), XP_001095740.1; wolf (Canis), XP_545648.2; chicken (Gallus), NP_001006590.1; zebrafish, (Danio) NP_571055.1; frog (Xenopus), XP_002934004.2.


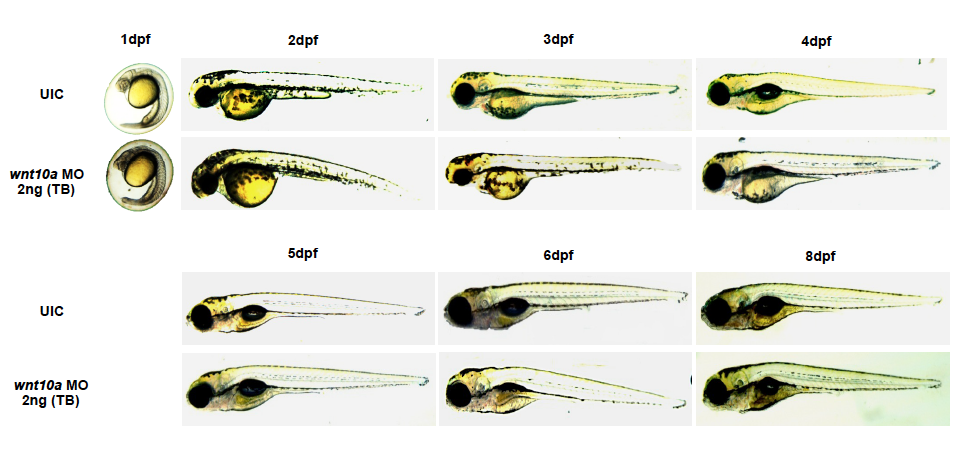


**Supplementary Fig. 2. Analysis of Zebrafish *wnt10a* Morphant Embryos.** *wnt10a* MO-injected fish showed similar body structure and appearance compared to uninjected fish. Developmental periods were classified based on the landmarks: segmentation period (10-24 h), pharyngula period (24-48 h), hatching period (48-72 h), and larval (3-29 days).


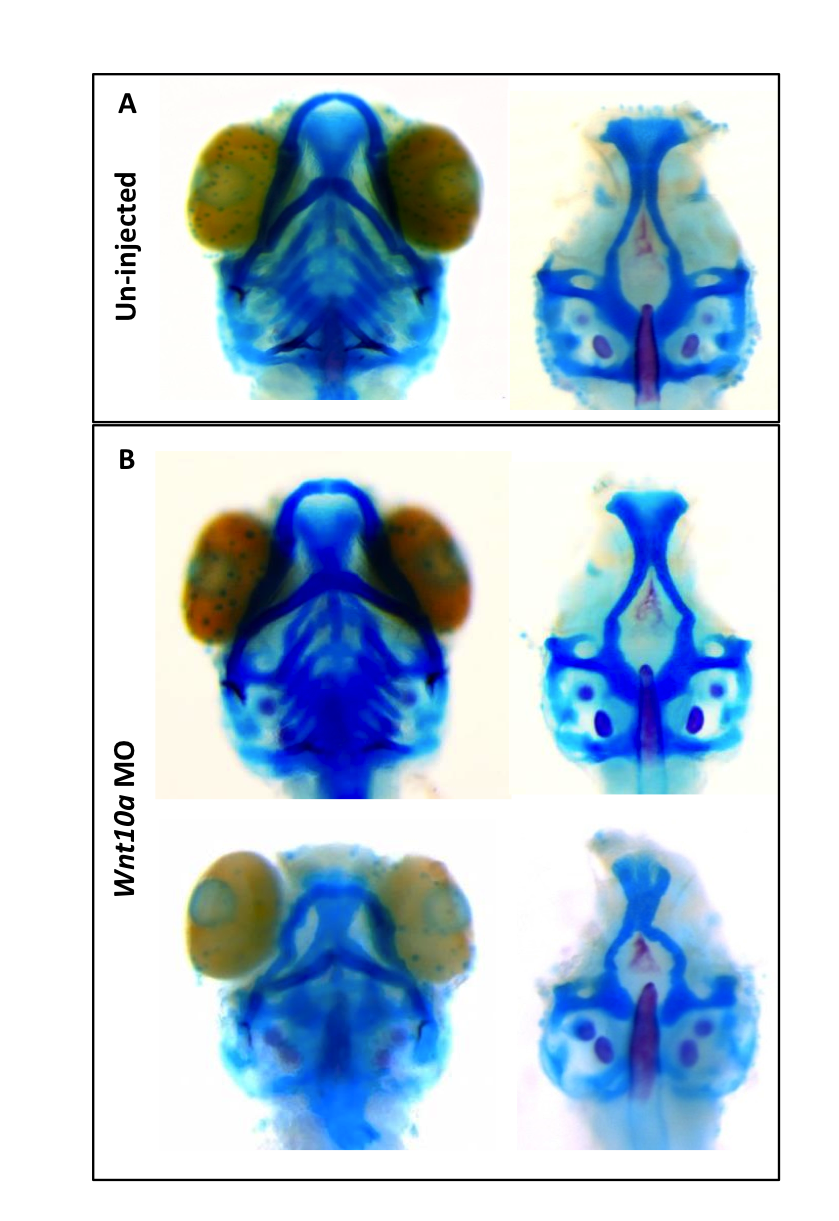


**Supplementary Fig. 3. *wnt10a* Knockdown Causes Cartilage Abnormalities in Zebrafish.** Alcian blue and alizarin red staining of (A) Un-injected and (B) *wnt10a* MO-injected zebrafish at 5dpf. While un-injected fish have no noticeable cartilage defects, MO-injected fish present with a range of mild (top) to more severe (bottom) cartilage abnormalities.

**Supplementary Table 1.** *WNT10A* custom-SNPs used for genotyping.

| **Nucleotide change** | **Amino acid** | **dbSNP Id.^*^** | **MAF ^#^** | **SIFT** | **Polyphen** | **VIPUR** |
| --- | --- | --- | --- | --- | --- | --- |
| c.149C>T | Pro50Leu | rs199980023 | 0.0001 | 0.01 | 0.617 | --- |
| c.321C>A | Cys107* | rs121908119 | 0.001 | 0 | 0.996 | --- |
| c.487C>T | Arg163Trp | rs368280129 | N/A | 0.01 | 0.91 | .623 |
| c.511C>T | Arg171Cys | rs116998555 | 0 | 0 | 0.70 | .355 |
| c.637G>A | Gly213Ser | rs147680216 | 0 | 0 | 0.994 | .91 |
| c.682T>A | Phe228Ile | rs121908120 | 0.02 | 0 | 0.994 | .914 |
| c.826T>C | Cys276Phe | rs201593552 | 0 | 0 | 0.998 | .971 |
| c.1070C>T | Thr357Ile | N/A | N/A | 0 | 1 | .80 |
| c.1690G>C | Asp353His | rs371998817 | N/A | 0.01 | 0.964 | .793 |
| c.1711C>A | Arg360Cys | COSM1530886 | N/A | 0 | 0.997 | .96 |

^*^ Custom-made SNP assays, Applied Biosystems.

^#^ MAF, minor allele frequency, in CEU or EUR populations per 1000genomes project database.

**Supplementary Table 2.** Morpholino and primer sequences used in this study.

| **Morpholinos** | **Sequences** |
| --- | --- |
| Mismatch control MO (MM MO) | 5’-GTCaTaAGAaCTCATTgATaGAATC-3’ |
| Translational blocking MO (Tb MO) | 5’-GTCGTGAGAGCTCATTCATGGAATC-3’ |
| Splicing blocking MO (SB MO) | 5’-CTGTTTGATTTGATCGCTTACCCCT-3’ |
| **Primers** | **Sequences** |
| *wnt10a* | Cat. No. QT02422308 (Qiagen) |
| *dlx2b* | Forward: 5'-ACGATTTAGGCTATGCTTCCTC-3'  Reverse: 5'-GCTTTCCATTCACCATGCG-3' |
| *msx1* | Forward: 5'-TCACACCCGTTTCACAGAC-3'  Reverse: 5'-CGGCAAACTTCACAAGTCAC-3' |
| *pax9* | Forward: 5'-TCAATGGTGTGGATAAGCCTC  Reverse: 5'-CATGTAGGGTGACACTTGGG |
| *axin2* | Forward: 5'-TTGGAAGATCGGGACTTTACG-3'  Reverse: 5'-AGTGTCCTATTCATGGCTCTTG-3' |
| *eda* | Forward: 5'-CTCAAACGCGAAATATCCCAG-3'  Reverse: 5'-GTGTTCATCCCTCCATCTGC-3' |
| *actb1* | Cat. No. QT02174907 (Qiagen) |

**Supplementary Table 3.** Number of embryos with and without teeth.

| **MO** | **Total**  **injected** | **24hpf**  **alive** | **5dpf**  **alive** | **Total**  **Post fixation & Staining** | **Teeth +** | **Teeth -** |
| --- | --- | --- | --- | --- | --- | --- |
| **Tb MO 2ng** | 40 | 40 | 38 | 35 | 11% (4/35) | 79% (31/35) |
| **Sb MO 2ng** | 98 | 66 | 64 | 61 | 3%  (2/61) | 96% (59/61) |
| **Mismatch MO 2ng** | 92 | 80 | 74 | 73 | 96%  (70/73) | 4%  (3/73) |
| **UIC** | 85 | 65 | 64 | 64 | 100% (64/64) | 0%  (0/64) |

Tb MO, translation blocking morpholino

Sb MO, splice blocking morpholino

UIC, uninjected control

**Supplementary Table 4.** PCR primers used for *wnt10a* overexpression and analysis of mutant alleles.

| **Primer** | **Direction** | **Variant** | **Sequence (5’ to 3’)** |
| --- | --- | --- | --- |
| Dr wnt10a G248S | Forward | G248S* | GGACTCCTGGGAGTGGGGCAGCTGCAGTCCCAACGTGGAG |
|  | Reverse |  | CTCCACGTTGGGACTGCAGCTGCCCCACTCCCAGGAGTCC |
| Dr wnt10a T382I | Forward | T382I* | GGGTCGGACTCTGCCGGGATCCAAGGCCGGATCTGTAACA |
|  | Reverse |  | TGTTACAGATCCGGCCTTGGATCCCGGCAGAGTCCGACCC |
| Hs WNT10A G213S | Forward | G213S | GGACTCCTGGGAGTGGGGCAGCTGCAGCCCCGACATGGGC |
|  | Reverse |  | GCCCATGTCGGGGCTGCAGCTGCCCCACTCCCAGGAGTCC |
| Hs WNT10A T357I | Forward | T357I | CGCCTGGACTCGGCCGGCATCGTGGGCCGCCTGTGCAACA |
|  | Reverse |  | TGTTGCACAGGCGGCCCACGATGCCGGCCGAGTCCAGGCG |

* G248S and T328I are corresponding zebrafish mutant alleles for human G213S and T357I respectively
